# Adaptation with transcriptional regulation

**DOI:** 10.1038/srep42648

**Published:** 2017-02-24

**Authors:** Wenjia Shi, Wenzhe Ma, Liyang Xiong, Mingyue Zhang, Chao Tang

**Affiliations:** 1 Center for Quantitative Biology, Peking University, Beijing 100871, China; 2 Department of Systems Biology, Harvard Medical School, Boston, Massachusetts, United States of America; 3 School of Physics, Peking University, Beijing 100871, China; 4 Peking-Tsinghua Center for Life Sciences, Peking University, Beijing 100871, China

## Abstract

Biochemical adaptation is one of the basic functions that are widely implemented in biological systems for a variety of purposes such as signal sensing, stress response and homeostasis. The adaptation time scales span from milliseconds to days, involving different regulatory machineries in different processes. The adaptive networks with enzymatic regulation (ERNs) have been investigated in detail. But it remains unclear if and how other forms of regulation will impact the network topology and other features of the function. Here, we systematically studied three-node transcriptional regulatory networks (TRNs), with three different types of gene regulation logics. We found that the topologies of adaptive gene regulatory networks can still be grouped into two general classes: negative feedback loop (NFBL) and incoherent feed-forward loop (IFFL), but with some distinct topological features comparing to the enzymatic networks. Specifically, an auto-activation loop on the buffer node is necessary for the NFBL class. For IFFL class, the control node can be either a proportional node or an inversely-proportional node. Furthermore, the tunability of adaptive behavior differs between TRNs and ERNs. Our findings highlight the role of regulation forms in network topology, implementation and dynamics.

Current biology has moved into a quantitative era. Mathematical models are increasingly used in researches to help elucidating underlying mechanisms of biological processes. Among different models, the biological network is a natural way to represent complicated biological regulations, and straightforward to translate into a mathematical model. One interesting feature of network model is that the network topology and network function are related. For example, bistable^1^,^2^ and excitable systems^3^ often have positive feedback loops whereas oscillating systems often come with negative feedback loops^4^,^5^. The relationship has significant meaning to biology research because it shines light on understanding the complex regulation diagrams and predicts new regulations. Our previous study has shown that there is a relationship between biological function and network topology but it is not one-to-one mapping, instead, a small set of different network topologies can lead to the same function. For example, the adaptation networks has either negative feedback loops or incoherent feedforward loops^6^. Other groups have also shown similar properties of function-topology relationship in other biological systems^7^,^8^,^9^,^10^,^11^,^12^,^13^. One remaining question of this kind of study is whether specific regulation forms or rules could change the function-topology relationship. Different reactions such as phosphorylation, degradation and gene regulation have different time scales and regulation characteristics. Mathematically, different regulations are represented with different function forms. For example, enzymatic reactions follow Michaelis-Menten forms while gene regulations follow Hill functions.

Adaptation exists in a broad range of biological systems. Typical examples of adaptation include the adaptation of signal transduction pathway^14^,^15^, adaptation of neuron activity^16^,^17^, stress response^18^,^19^,^20^, bacteria chemotaxis^21^,^22^,^23^ and homeostasis^24^. In this work, we still use adaptation as the model system to study function-topology relationship of biological networks. A typical adaptation process contains two parts, a pulse phase indicating the sensing of stimulus, where we define a quantity- response to represent it, and a recovery phase indicating adaptation to the environment, where adaptation error is defined (Fig. 1A). Perfect adaptation is achieved if the output of the system recovers to the exact original value before stimulation. In our previous work, by investigating the whole three-node ERNs, we found two classes of network topologies that are capable of perfect adaptation: a negative feedback loop with a buffer node (NFBLB) and an incoherent feedforward loop with a proportioner node (IFFLP)^6^. Both of these two classes have an intermediate node which serves as a controller to determine the mechanism of adaptation. In the NFBLB class, the intermediate node is an integral feedback controller which buffers the change of output by integrating the error of the output node. In the IFFLP class, the intermediate node is a proportioner which balances the influence of the input on the output by responding to input signal proportionally (Fig. 1C).

Gene transcriptional expression changes in cellular adaptation to short- or long-term environmental changes, with extensive regulation occurring at the transcriptional level^25^,^26^. So here, we used the framework of enzymatic adaptation^6^ to study the general design principles of gene regulatory networks for adaptation and explored the differences affected by regulatory rules. We studied three different types of transcriptional regulation logics. For all the rules, we obtained the same two general classes of adaptive core topologies: negative feedback loops (NFBLs) and incoherent feedforward loops (IFFLs) as in the ERNs. However, there are important differences between the adaptive TRNs and ERNs, in terms of how the control node gets involved and tuning of the response.

## Methods

### Model Construction

Gene expressions are regulated by other genes’ products that work as transcription factors (TFs). Commonly more than one TF binds to the respective gene regulatory sequences^27^, with the involvement of RNA polymerase complex, to determine the transcriptional activity. Simple logic model can be used to model multiple TF regulations^28^,^29^,^30^,^31^. In the following text, in characterizing a transcriptional network topology, every node stands for the transcriptional gene product and each link represents a transcriptional regulation by the gene product (as a TF) from which the link originates. The transcription activity is a function of the concentration of TFs. In equation (1), *f*_*x*_ or 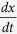 denotes the rate of concentration change of the gene product. We assume that gene activation is regulated while all the gene products undergo unregulated degradation. *G*_*x*_ is a function of all TF regulatory terms *g*, with the Hill function 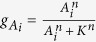 and 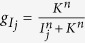 denoting the *i*th activation and *j*th repression terms on gene *x. v*_*x*_ is the maximal production rate of gene *x. τ*_*x*_ is the half-life of this gene product.


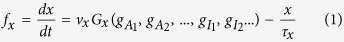


**Figure 1:**
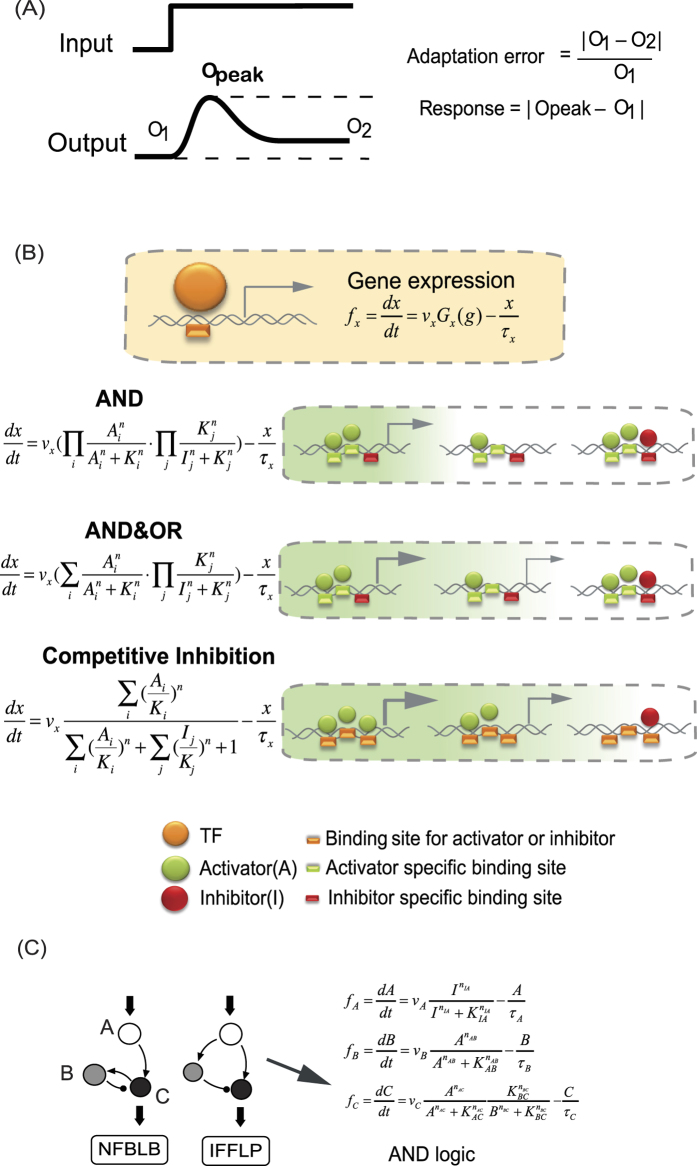
(**A**) Functional characterization of adaptation used in this study. (**B**) Models of transcriptional regulation with different logics. (**C**) Two families of perfectly adaptive networks among ERNs. The node A (white circle) and C (black circle) act as the input and output node, respectively. The node B (grey circle) is a control node which plays the role of an integrator in the NFBLB family and a proportioner in the IFFLP family. Right side is the transcriptional regulation model of IFFLP with AND logic.

Here we considered three logics for multiple TFs (Fig. 1B): (1) AND logic, where gene expressions are only turned on when all the activators are at high concentrations and the repressors are at low concentrations; (2) AND&OR logic, in which turning on any of the activators is enough to turn on the downstream genes, provided that the repressors are low; (3) Competitive Inhibition logic, in which the relative weight of repressor and activator together determines the downstream gene activation. Here, activators and repressors compete for the same binding sites, and the repressors decrease the effect of activators instead of blocking gene expression. The IFFLP modeled with AND logic is shown in Fig. 1C as an example.

## Results and Discussion

### Computationally Searching for Circuits Capable of Adaptation with Transcriptional Regulation

Searching for adaptive topologies can be achieved by two complementary approaches. One approach is computational enumeration, which is feasible for relatively small-size networks. The other one is theoretical analysis around the system’s steady state, which can give rigorous conditions for perfect adaptation.

We firstly computationally searched all possible networks with three nodes: the input (A), the output (C), and the control (B) nodes. Each node can regulate three nodes (two other nodes and self), so that each network contains up to nine links (positive, negative or none regulation). We have 16038 networks in total (there are 19683 topologies in the whole three-node network space, but the topologies that have no direct or indirect links from the input to the output are excluded). Each node has a maximal production rate *v* and a decay rate *τ* as parameters, and each regulatory link has a Hill coefficient *n* and an activation/repression threshold *K* as parameters. For each network, 10,000 sets of parameters and three transcriptional regulatory logics are used in the ordinary differential equations (ODEs) simulation. During our simulations, an architecture is referred as a functional solution of perfect adaptation when it has: adaptation error <0.005, response >0.2 (Input changes from 0.06 to 0.6). The main results do not change with different criteria for function (Fig. S1).

### Adaptive TRNs Have Different Features and Parameter Constraints from ERNs

Our first question is whether all the functional solutions also converge on the NFBLB and IFFLP families. We separated the functional solutions of all three logics into two families: the NFBL family, which includes all solutions that contain a NFBL but no IFFL; the remaining family, the rest topologies in functional solutions except for the NFBL family. Interestingly, the topologies in the remaining family all contain the skeleton of an IFFL. Thus the core families to achieve adaptation both in TRNs and ERNs are all NFBLs and IFFLs. However, when we looked into more topological details, we found there are also some differences.

**Figure 2:**
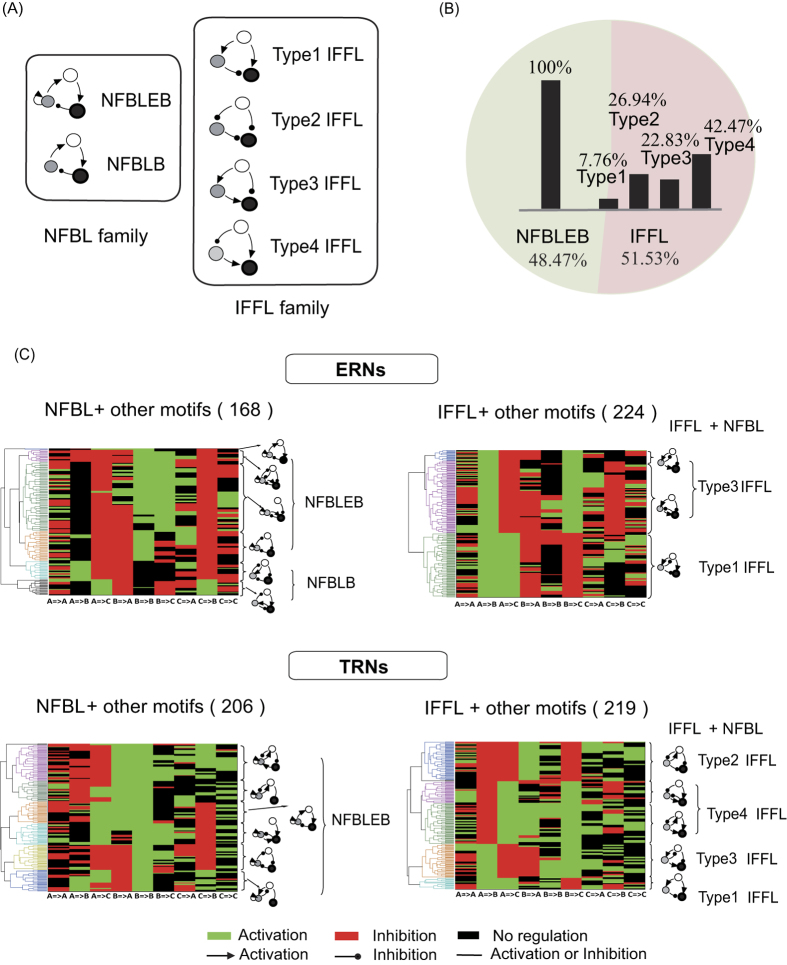
Comparison of adaptive networks with gene regulation and enzymatic regulation. (**A**) Categories of adaptive motifs. (**B**) Motifs compositions of adaptive solutions in TRNs. Bars in green and pink backgrounds are for NFBL and IFFL families, respectively. (**C**) Clustering results of adaptive TRNs (AND logic) and ERNs. The network motifs associated with each of the sub-clusters are shown on the right.

We analyzed the simulation results under AND logic as an example. 425 out of 16,038 topologies are functional, including 206 (48.47%) topologies belonging to the NFBL family and 219 (51.53%) the IFFL family (Fig. 2A and B). We separately clustered the networks from these two families using Hamming distance between architectures (Fig. 2C). Each column represents one specific regulation, and each row represents one architecture. The motifs extracted from each sub-cluster (listed on the right of each panel) indicate that: IFFLs work as a core structure and are very tolerable on additional regulations, and all the topologies in the NFBL family contain an auto-activation loop on regulatory node B (Fig. 2C).

To clearly figure out the differences between adaptive networks among TRNs and ERNs respectively, we then compared our clustering results with the simulation of ERNs (data from Ma *et al*., Fig. 2C), we found:

1. The minimal solutions contain three links within three nodes. That is to say, neither one-node nor two-node network is capable of performing adaptation in both these two regulatory conditions.
2. NFBLs and IFFLs are two families that can achieve adaptation in both these two regulatory conditions.
3. An auto-activation on the buffer node in the NFBL family is necessary for TRNs but optional for ERNs. This auto-activation results in a special kind of adaptive network: negative feedback loop with an exponential buffer node (NFBLEB) which helps NFBLs buffer the adaptation error in a logarithmic way^6^ (detailed example can be seen in next section). Meanwhile, all the negative feedback loops in both these two regulation conditions go through the buffer node rather than feedback from the output node to the input node directly.
4. All four types of IFFLs are adaptive in TRNs (Fig. 2C), while in ERNs there are only two (type 1 and 3) that are adaptive.

The simulation results of the other two transcriptional logics agree with the first 3 conclusions above, but differ in the 4th one: AND&OR logic has 3 types of adaptive IFFLs and Competitive Inhibition logic has only two types of adaptive IFFLs as in the enzymatic regulation (Fig. S2).

### Mechanisms for Transcriptional Adaptation

Distinct topological features between TRNs and ERNs lead us to investigate the origin of these differences analytically. We addressed this question by performing a linear analysis of the transcriptional systems. Theoretically, the equations for any three-node network *dA*/*dt* = *f*_*A*_(*A, B, C, I*), *dB*/*dt* = *f*_*B*_(*A, B, C*), and *dC*/*dt* = *f*_*C*_(*A, B, C*) can be linearized around their steady state A^*^, B^*^, and C^*^ (provided that the system has a steady state). The deviation Δ*A,* Δ*B* and Δ*C* from the steady state, when the input changes from *I* to *I* + Δ*I*, satisfy the following linearized equation:


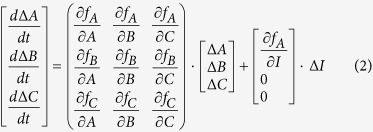


**Figure 3:**
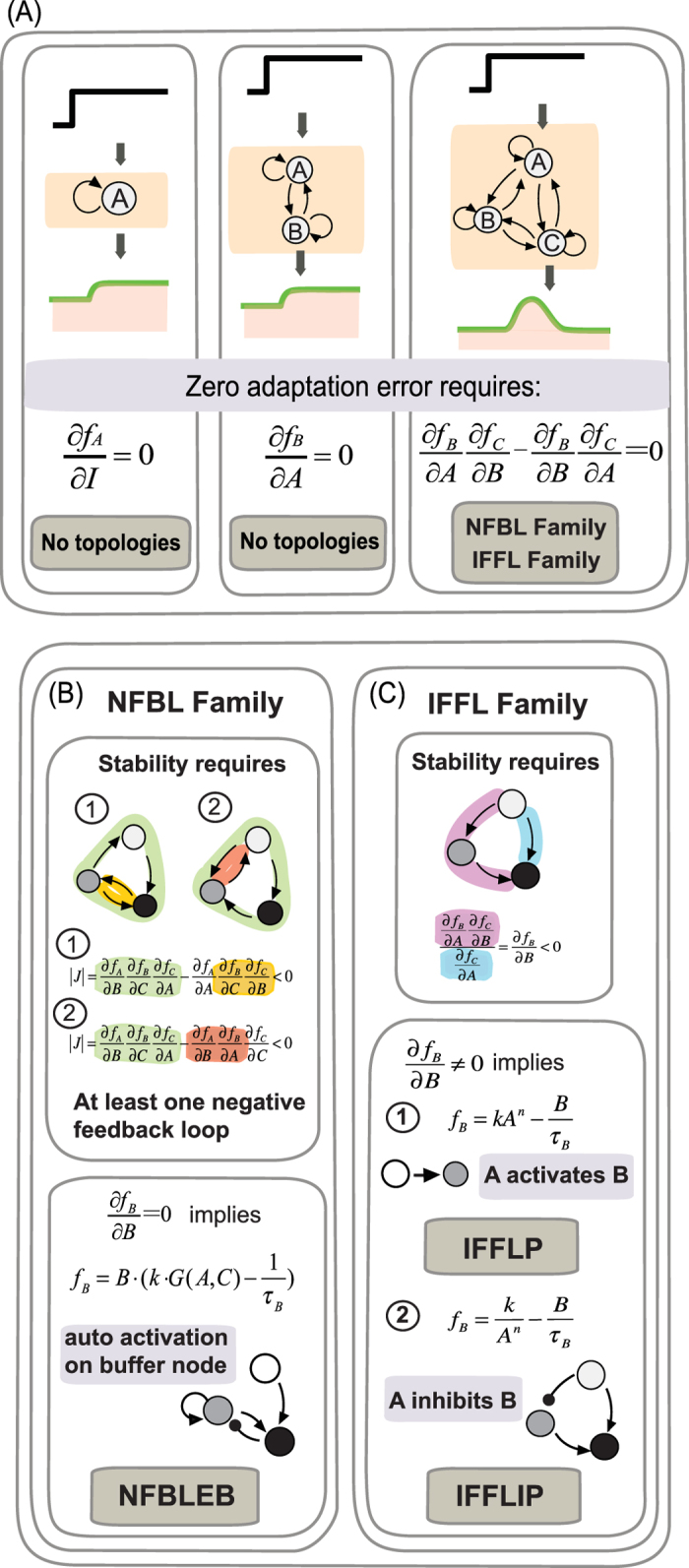
Theoretical analysis of TRNs. (**A**) The requirement of zero adaptation error around a stable steady state results in the equations that are shown for one-, two- and three-node networks. One- and two- node systems cannot satisfy the requirement, so there are no topologies to achieve adaptation in these two systems. Two kinds of three-node topologies can satisfy the requirement: NFBL and IFFL. (**B**) The NFBL family achieves perfect adaptation with ∂*f*_*B*_/∂*B* = 0 and the Jacobian determinant |J| < 0 (stability requirement). With the condition ∂*f*_*B*_/∂*B* = 0, the terms in the determinant |J| correspond to different feedback loops as colored in the figure. So at least one NFBL is required in this family. Two NFBLs would result in a more negative |J|, which can lead to a smaller adaptation error. No feedforward loop can be present in this family. The condition ∂*f*_*B*_/∂*B* = 0 can be satisfied in the TRN model with the buffer node B auto-activating itself with Hill coefficient 1. (**C**) The IFFL family achieves perfect adaptation with ∂*f*_*B*_/∂*B* ≠ 0 and also |J| < 0, which implies ∂*f*_*B*_/∂*B* < 0. For this family, the links colored in the figure are necessary to be present and constitute an IFFL. Two opposing regulations on C need to be cancelled out which requires certain input-independent relationship between A and B at their steady state. The proportionality relationship with A activating B or inverse proportionality relationship with A inhibiting B can be established by the equation of node B.

The requirement for perfect adaptation is ΔC^*^  =  0, which means that after the input change the output value exactly returns to its original state. All the topologies satisfied the requirement fall into two classes that both have three nodes within (Fig. 3): (1) NFBLs with ∂*f*_*B*_/∂*B* =  0. At least one NFBL is required in this family (colored loop in Fig. 3B NFBL family); and (2) IFFLs with ∂*f*_*B*_/∂*B* < 0 (colored loop in Fig. 3B IFFL family)^6^. These conclusions have no restrictions on the specific form of the system’s function except that they should have stable steady states (see Supplementary materials for one and two- node system’s derivations). Thus the B node equation (equation (3)), which we denote as B-equation, plays a very important role for achieving perfect adaptation as ∂*f*_*B*_/∂*B* is the key point. The mathematical form of B-equation determines how the system achieves the requirement of perfect adaptation both in topological design and parameter constraints.


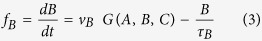


#### Exponential Buffer Node in the Negative Feedback Loop

In B-equation (equation (3)), there is a linear decay term. For NFBLs, the condition for perfect adaptation requires ∂*f*_*B*_/∂*B* = 0, which can be satisfied robustly if the production term of B-equation also contains the variable B so that it can be factored out:


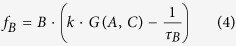


**Figure 4:**
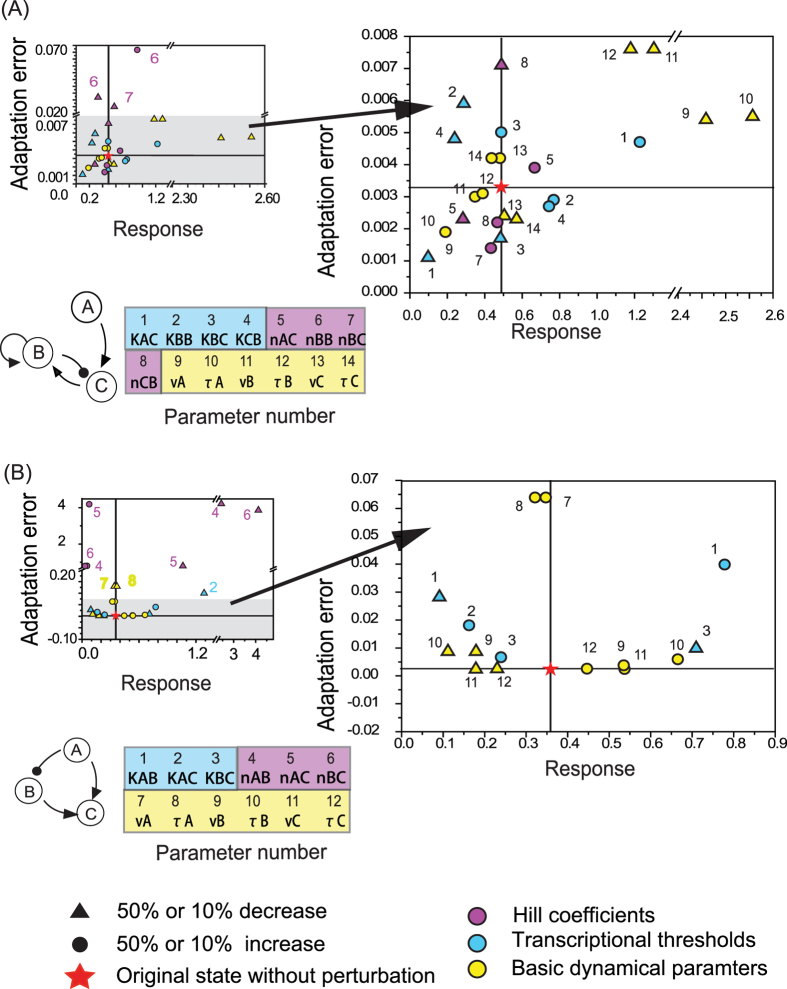
The amplitude of response changes with parameters’ perturbations in two adaptation motifs. Each dot shows the result with a perturbation of the corresponding numbered parameter (circle and triangle for increase and decrease respectively). The performance of the system without perturbations is marked with the red star. Parameters are grouped into colored groups. Purple, blue and yellow for Hill coefficients, transcriptional thresholds and basic dynamic parameters, respectively. (**A**) The NFBLEB system. *n*_*BB*_ is perturbed with 10% and other parameters with 50% increase and decrease. (**B**) The IFFLIP system. All the parameters are perturbed with 50% increase and decrease.

The way to achieve this is to have node B positively regulating itself with *n*_*BB*_ = 1 and *B* ≪ *K*_*BB*_. As an example of NFBL (Fig. 4A) with AND logic, the ODEs of the system are:


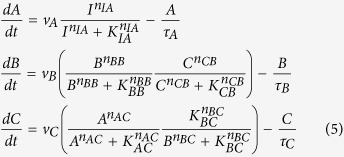


If the buffer node B works with *B *≪ *K*_*BB*_ and in a non-cooperating form (the Hill coefficient being 1, the rate equation for B can be approximated by:


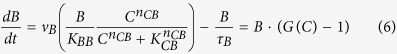


where *G(C*) is a function of only *C*. So in steady state G(C^*^) = 1 and C^*^ = constant, independent of the input. The node B integrates the relative difference between the output activity C and its input-independent steady-state value in a logarithm form: 
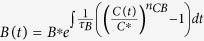
 (assuming *K*_*CB*_ ≫ *C* for simplicity). Here, node B plays the role of an exponential integrator of the adaptation error. All the adaptive NFBLs share this characteristic.

#### Inversely Proportional Node in the Incoherent Feedforward Loop

For transcriptional IFFLs, adaptation is achieved by a balance between the transcriptional production rate change caused by two signal-transmitting pathways acting on node C and the linear decay of C. Thus at steady state the production rate should maintain constant that is independent of the input to balance the unchanged decay term, which means the co-regulators of output, node A and B must establish certain relationship to satisfy the above requirement. In the three-node network, the B-equation undertakes the task to establish this relationship (Fig. 3C). In the case of A activating B, a robust proportional relationship can be established with the regulatory TF working with *A* ≪ *K*_*AB*_:


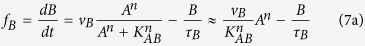


At steady state, *k*′A^*n^ = B^*^, where 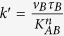. In the case that A inhibits B, a robust inversely proportional relationship can be established with A ≫ *K*_*AB*_:


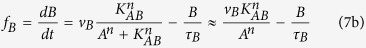


At steady state, A^*n^B^*^ = *k*′, where 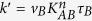. These relationships lead to incoherent feed-forward loops with a proportional node (IFFLP) or an inversely proportional node (IFFLIP). Here, the node B can be a proportioner or an inverse proportioner, whereas in enzymatic regulation, B can only be a proportioner in IFFLP^6^.

Let us analyze one specific IFFLIP in detail (Fig. 4B). Assuming the AND rule, the ODEs of the system are:


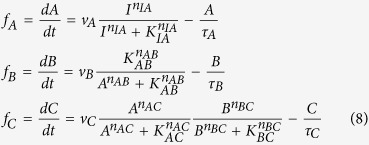


with *A* ≫ *K*_*AB*_, the equation of node B becomes:


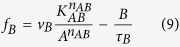


and thus at steady state we have:


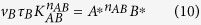


When the TFs A and B satisfy *A* ≪ *K*_*AC*_ and *B* ≪ *K*_*BC*_, we have:


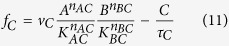


which, combining with equation (10), leads to a steady state solution of 
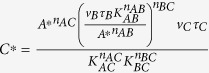
. C∗ is a constant, if


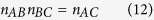


Note that the Hill coefficients do not have to be 1, as long as they satisfy equation (12), the system will adapt. This more relaxed condition on Hill coefficient differs from that of the adaptive IFFLPs with enzymatic regulation where the Hill coefficients have to be 1^6^.

#### Tuning Response of Adaptive TRNs

For an adaptive system, the transient response to an input change transduces information to the downstream molecules or pathways. Thus, the ability of tuning the response of an adaptive network is important. In the analysis above, we noticed that the adaptive NFBL and IFFL families have different parameter constraints both within TRNs and compared with ERNs. For the NFBLEB system, only the parameters in the auto-activation term in B-equation, *K*_*BB*_ and *n*_*BB*_ are critical for ensuring small adaptation errors. While for the IFFLIP system, constraints of more parameters are required to achieve adaptation. To investigate the tunability of the response for these two families, we performed a single parameter perturbation analysis. Specifically, we increased or decreased only one parameter each time by 20% or 50%, and monitored the adaptation behavior (Fig. 4). We (arbitrarily) grouped all the parameters into three groups: Hill coefficients *n* (purple), transcriptional thresholds *K* (blue), and the basic dynamic parameters (yellow), which include half-life *τ* and maximal production rate *v* (Fig. 4).

As can be seen from Fig. 4A, for the NFBLEB system, a small adaptation error can be maintained with respect to changes of many parameters, with the exception of *n*_*BB*_. We zoomed in those parameter changes that resulted in small adaptation errors (grey region in Fig. 4A, which is shown on the right on a different scale). The most efficient parameters for tuning the response are *τ*_*A*_ and *v*_*A*_. The decrease of *τ*_*A*_ and *v*_*A*_ decrease C, but also decrease the inhibitor B. The weaker inhibition from node B after the input change leaves more time for node C to increase transiently, thus contributes to a larger response. The second best group is *τ*_*B*_, *v*_*B*_ and *K*_*AC*_. Decreasing *τ*_*B*_ or *v*_*B*_ leads to a decrease in B, while an increase of *K*_*AC*_ slows down the signal transmission from node A to C and decreases B at the beginning. Through the decrease of B, these perturbations leave more time for the output to transiently increase and thus contribute to a larger response. The Hill coefficients can also alter the response by changing the reaction dynamics. For example, the increase of *n*_*AC*_ speeds up the transient increase of C, thus contributes to an increase of response. However, the Hill coefficient group shows weaker tunability than the other two groups. In summary, in this specific system, the concentration of the inhibitor B is sensitive to tune for the response. We rank the efficiency of tuning the response among parameter groups: *K*_*AC*_ > *K*_*CB*_ > *K*_*BC*_; *τ*_*A*_ > *τ*_*B*_ > *τ*_*C*_ (*v* and *τ* have similar effects). The more upstream in the signaling feedback loop, the more efficient to tune the response (In the loop A ->C->B->C, A is the first to receive the signal, B is the second one, C is the last one that needs to integrate A and B).

In Fig. 4B, for the IFFLIP system, a small adaptation error cannot be maintained with respect to changes of many parameters. We also zoomed in those parameter changes with relative smaller adaptation error for the IFFLIP system (grey region in Fig. 4B). The Hill coefficient group performs the worst because they should satisfy certain relationship mentioned in the previous section. Not all the transcriptional thresholds tolerate poorly in the perturbations, although they are all required to work in certain regions. Among them, *K*_*BC*_ is a potential adjuster that can maintain a small adaptation error and tune the response. The decrease of *K*_*BC*_ speeds up B’s activation on C and thus promote C’s transient increase (and vice versa). Meanwhile, owing to the inhibition by node A, B always maintains at low concentration, so it is easy for B to work with *B* ≪ *K*_*BC*_, which contributes to the tolerance of parameter perturbation for *K*_*BC*_. The basic dynamic parameters, especially *τ*_*B*_, *v*_*B*_, *τ*_*C*_ and *v*_*C*_ are efficient for tuning the response. Larger *τ*_*B*_ or *v*_*B*_ increases B, thus B waits for longer time to be inhibited by A till it reaches a low concentration and cannot activate node C. It leaves more time for C to transiently increase and leads to a larger response. *v*_*A*_ and *τ*_*A*_ behave poorly with perturbations at maintaining a small adaptation error, because A needs to satisfy both *A* ≫ *K*_*AB*_ (as an inhibitor of node B) and *A*  ≪ *K*_*AC*_ (as an activator of node C). In summary, fewer parameters in the IFFLIP systems performed well at tuning the response than the NFBLEB system. The Hill coefficient group is the worst option to tune. Tuning the binding affinity of TF B of gene C, or changing the half-lives or maximal transcriptional rate of protein B and C can improve the IFFLIP system’s response.

### The Roles of Transcription Logic

**Figure 5:**
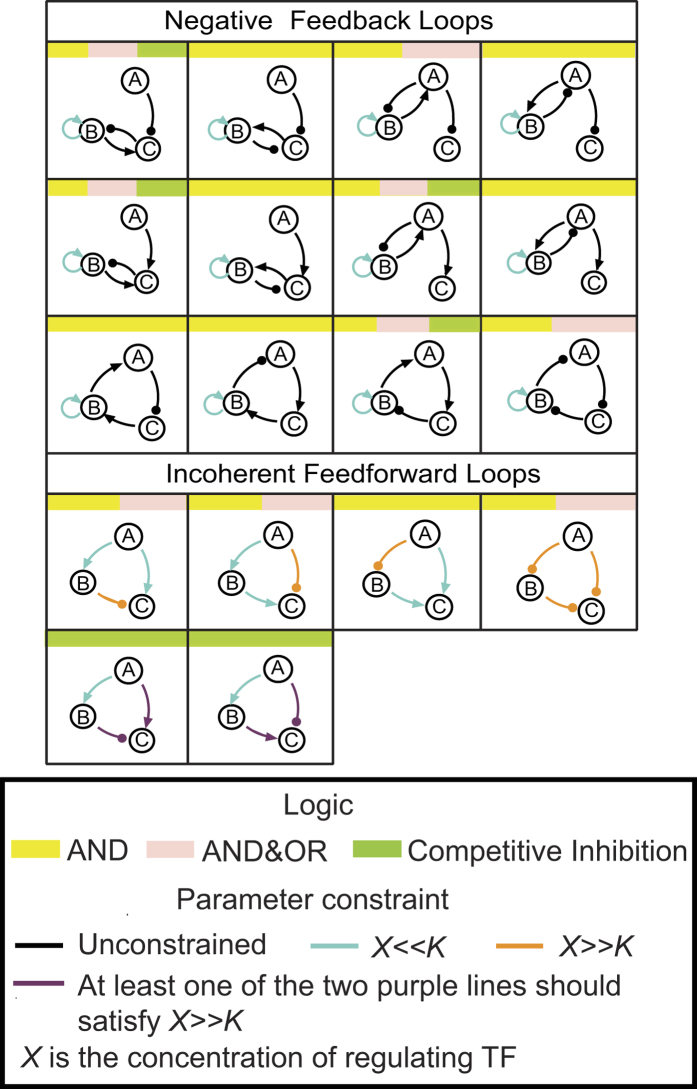
Minimal topologies of perfect adaptation with three regulatory logics. Yellow, pink and green tags represent AND, AND & OR and Competitive Inhibition logics, respectively. Colored lines represent constraints on the parameters as indicated in the legend.

When a node is transcriptionally regulated by more than one link, different transcription logics have different mathematical forms, which may have different consequences on the topological requirement for adaptation. Following the theoretical analysis, we derived the minimal design table with three transcriptional logics (Fig. 5). Every minimal topology is labeled with yellow, pink and green tags representing its feasibility in AND, AND&OR, and Competitive Inhibition logics, respectively. There are 12 NFBLs and 4 IFFLs in total, but not all of them are feasible for all logics.

For the NFBL family, there is one common feature that all NFBLs have an auto-activation of the buffer node B working with *B* ≪ *K*_*BB*_ and *n*_*BB*_ = 1.12 NFBLs are all capable of achieving perfect adaptation with the AND logic. For the AND&OR and Competitive Inhibition logics, no activation on node B other than the auto-activation is allowed. This is because with these logics (Fig. 1B), it is hard to factor out variable B with two or more activation terms in the B-equation. For the Competitive Inhibition logic, the requirement that each node should have at least one activator (Fig. 1B) further reduces the number of feasible topologies.

For the IFFL family, there are total of 4 topologies (Fig. 5). All 4 IFFLs are adaptable for the AND logic, two of which with proportional mechanism and the other two with inversely-proportional mechanism. For the AND&OR logic, node C can have at most one activating regulation, otherwise the summation of regulations from nodes A and B are hard to cancel out. This leaves 3 IFFLs for this logic. For the Competitive Inhibition logic, the simplest way to achieve a constant output is to establish a linear relationship between *A*^*n*^ and *B*^*n*^ from B-equation (the Hill coefficient n can be 1), and then to have the two nodes A and B regulating C oppositely (one activating and one inhibiting). In the region *A* ≫ *K*_*AC*_ and/or *B* ≫ *K*_*BC*_, the regulations from nodes A and B cancel out, making C a constant at steady state. This scenario only works with the proportional mechanism, so only two IFFLs are feasible for perfect adaptation with the Competitive Inhibition logic (Fig. 5 and see Supplementary materials for detailed derivations).

## Discussion

Generating a comprehensive function-topology map can supply a complete design table as well as illustrate the underlying mechanism to achieve the function^32^,^33^. Nature provides a versatile toolbox for biochemical reactions and regulations. In this study, we focused on a well-studied function, perfect adaptation to investigate the consequence of different regulation types and rules on topology, parameters constraints, and other functional features. We found that similar to the enzymatic networks, the topologies of the transcriptional adaptive networks belong to two general classes: negative feedback loop (NFBL) and incoherent feed-forward loop (IFFL). However, there are several distinct features for the adaptive TRNs. First, an auto-activation loop of the buffering control node with Hill coefficient 1 is necessary for the NFBL class. This is more restrictive compared with the adaptive ERNs of the NFBL class in which this loop is optional. The reason behind the auto-activation loop is that in TRNs there is always a (linear) decay term in each rate equation, including the one for the control node B. In order for the NFBL motif to satisfy the adaptation condition ∂*f*_*B*_/∂*B* = 0, the activation term in the rate equation should also contain a linear factor in B. Whereas, in ERNs the switch from activated to inactivated state can be done by other enzymes, so it is not necessary to have an auto-activation loop on B. On the other hand, there are more distinct topologies available for the adaptive TRNs of the IFFL class, in comparison to that of ERNs. For TRNs, the control node can be either a proportional node or an inversely-proportional node, although the tunability of the response in the IFFLIP class is quite limited. Adaptive TRNs also have fewer restrictions on Hill coefficients.

Biological systems often respond to signal changes in multiple time scales, e.g. with fast reactions at the beginning and changes in gene expression at later stages^34^. In the budding yeast *Saccharomyces cerevisiae*, when facing with osmotic stress, cells adapt through the accumulation of glycerol^35^. They first close glycerol channels and rearrange metabolic activities in cytosol within minutes to promote glycerol accumulation, and then express more than 300 genes, including the cytosolic glycerol synthesis genes, GPD1 and GPD2, at a time scale about 30 minutes and longer^36^. This adaptation process involves both enzymatic and transcriptional regulations^34^,^37^,^38^. Previous studies focused on the enzymatic regulations^39^,^40^, while it remains unclear what the role of transcription in this adaptive system is. Our study may provide some clues for transcriptionally involved adaptation systems.

From a synthetic biology point of view, type1 IFFL circuits with a proportional control node have been constructed and shown to perform adaptation^41^,^42^. It would be interesting to see if IFFLIP circuits can also be constructed and achieve the desired function.

## Additional Information

**How to cite this article**: Shi, W. *et al*. Adaptation with transcriptional regulation. *Sci. Rep.* **7**, 42648; doi: 10.1038/srep42648 (2017).

**Publisher's note:** Springer Nature remains neutral with regard to jurisdictional claims in published maps and institutional affiliations.

## Supplementary Material

Supplementary Information
