# Supplementary material for: Adaptation with transcriptional regulation
Source: Sci Rep. 2017 Feb 24;7:42648. doi: 10.1038/srep42648 (PMC5324054; doi:10.1038/srep42648)
Supplement: Supplementary Information [file srep42648-s1.pdf]

Supplementary Materials

Adaptation with Transcriptional Regulation

Wenjia Shi, Wenzhe Ma, Liyang Xiong, Mingyue Zhang, and Chao Tang

A. Theoretical derivations for one- and two- node adaptive networks

We verified that one-node systems and two-node systems with input and output on separate nodes cannot adapted perfectly. However, two-node networks with input and output on the same node are capable of perfect adaptation in particular topologies and parameter constraints (Zhang *et al.*, in preparation).

For one-node systems, the time derivative of gene expression is a function of the concentration of input and the node itself $\frac{dA}{dt} = f_A(A, I)$. The linearized equation is given below:

$$\frac{d\Delta A}{dt} = \frac{\partial f_A}{\partial A}\Delta A + \frac{\partial f_A}{\partial I}\Delta I \tag{1}$$

Thus adaptation error is:

$$\varepsilon = \frac{\frac{\Delta A^*}{A^*}}{\frac{\Delta I}{I}} = \frac{\frac{I}{A^*}\cdot\frac{\partial f_A}{\partial I}}{\frac{\partial f_A}{\partial A}} \tag{2}$$

$\frac{\partial f_A}{\partial I} = 0$ at steady state should be satisfied if the adaptation error is zero (perfect adaptation), which means that no input signal is transmitted to the downstream system. So the one-node system cannot achieve perfect adaptation.

For two-node systems (with an input node A, an output node B), the adaptation error is captured by equation 3:

$$\varepsilon = \frac{\frac{\Delta B^*}{B^*}}{\frac{\Delta I}{I}} = \frac{\frac{\partial f_B}{\partial A}\cdot\frac{\partial f_A}{\partial I}}{J}\cdot\frac{I}{B^*} \tag{3}$$

The system needs to satisfy $\frac{\partial f_B}{\partial A} = 0$ at steady state to achieve zero adaptation error, which means that the input node does not regulate the output node. Thus the two-node systems cannot achieve perfect adaptation if the signal is to be transmitted from A to

B.

B. Enumeration of Three-node Topologies and simulation parameters

In the enumeration part, we normalized equations following our previous methods [1], which can reduce the parameters space (for example, the equation (4) is normalized to equation (5). For each network topology, 10,000 parameter sets were sampled uniformly in logarithmic or linear scale using the Latin hypercube sampling method [2]. The sampling ranges of the parameters are $K \sim 0.001-1$ (sampled in logarithmic scale) $n \sim 1-4$ (sampled linearly) and $\tau \sim 1-100$ (sampled in logarithmic scale). For AND&OR logic, where all the activation terms are summed up, we averaged all the activation terms in the simulation to ensure the normalization.

$$\frac{dA}{dt} = V_A \frac{B^n}{B^n + k_{BA}{}^n} - \frac{A}{\tau_A} \tag{4}$$

$$\frac{dA}{dt} = \frac{1}{\tau_A}\left(\frac{B^n}{B^n + k_{BA}{}^n} - A\right) \tag{5}$$

The simulation parameters in Fig. 4 in maintext are list below:

(1) For the NFBLEB system, $K_{IA} = 0.4, n_{IA} = 1, v_A = 0.5, \tau_A = 1.756993,$ $n_{BB} = 1,$ $K_{BB} = 9.172191,$ $K_{CB} = 0.580298,$ $n_{CB} = 2.835694, v_B = 1, \tau_B = 22.631118,$ $K_{AC} = 0.141219,$ $n_{AC} = 2.472565,$ $K_{BC} = 0.022553, n_{BC} = 1.733005,$ $v_C = 3,$ $\tau_C = 3.118057$.

(2) For the IFFLIP system, $K_{IA} = 0.4, n_{IA} = 1, v_A = 0.2, \tau_A = 5,$ $K_{AC} = 3,$ $K_{AB} = 0.02, n_{AB} = 2, v_B = 0.05, \tau_B = 35,$ $n_{AC} = 2, K_{BC} = 3, n_{BC} = 1, v_C = 80, \tau_C = 25$.

C. Simulation solutions for adaptive networks with AND&OR and Competitive Inhibition logics.

For AND&OR logic and Competitive Inhibition logic (Fig. S2), there are some NFBLs have extra positive regulations on node B from nodes other than itself. These results may be due to the simulation errors. For IFFL family, for AND&OR logic, theoretically, type 1, 3 and 4 can achieve adaptation and we see these motifs emerge in our simulation results (Fig. S2). Type 2 IFFL in which the node C is co-activated by node A and B, is not feasible to achieve perfect adaptation with AND&OR logic, while a part of the simulation results in IFFL family contains this motif as the skeleton. We find one possible explanation as the Fig. S3 shows. In this type 2 IFFL, the system's equations are:

$$
\begin{aligned}
f_A &= \frac{dA}{dt} = v_A \frac{I^{n_{IA}}}{I^{n_{IA}} + K_{IA}^{n_{IA}}} - \frac{A}{\tau_A} \\
f_B &= \frac{dB}{dt} = v_B \frac{K_{AB}^{n_{AB}}}{A^{n_{AB}} + K_{AB}^{n_{AB}}} - \frac{B}{\tau_B} \\
f_C &= \frac{dC}{dt} = v_C \left(\frac{A^{n_{AC}}}{A^{n_{AC}} + K_{AC}^{n_{AC}}} + \frac{B^{n_{BC}}}{B^{n_{BC}} + K_{BC}^{n_{BC}}}\right) - \frac{C}{\tau_C}
\end{aligned}
\tag{6}
$$

A and B establish an inversely proportional relationship through B-equation with $A >> K_{AB}$:

$$
f_B = v_B \frac{K_{AB}^{n_{AB}}}{A^{n_{AB}}} - \frac{B}{\tau_B} \tag{7}
$$

and $A^{*n_{AB}} B^{*} = k'$ at steady state ($k' = v_B K_{AB}^{n_{AB}} \tau_B$). Considering the simplest condition that $A << K_{AC}$ and $B << K_{BC}$, we have:

$$f_C = v_C\left(\frac{A^{n_{AC}}}{K_{AC}^{n_{AC}}} + \frac{B^{n_{BC}}}{K_{BC}^{n_{BC}}}\right) - \frac{C}{\tau_C} \tag{8}$$

which means the concentration of C is a function of the sum of two addends of A's concentration (assume all the Hill coefficients in equation (8) are 1, k_1, k_2 are parameters):

$$f_C = k_1 A + \frac{k_2}{A} - \frac{C}{\tau_C} \tag{9}$$

Fig. S3 shows a curve of the concerntration of C varies with A. There is a pit, which means A can vary in a region (light green region) where C maintains almost the same (light red region). In this condition, the output can maintain almost constant in simulation while A varies with input change.

While for the IFFL family with Competitive Inhibition logic, type 1 and 3 are feasible in theoretical analysis and also emerged in our simulation results.

D. Design table of simplest motifs with three logics

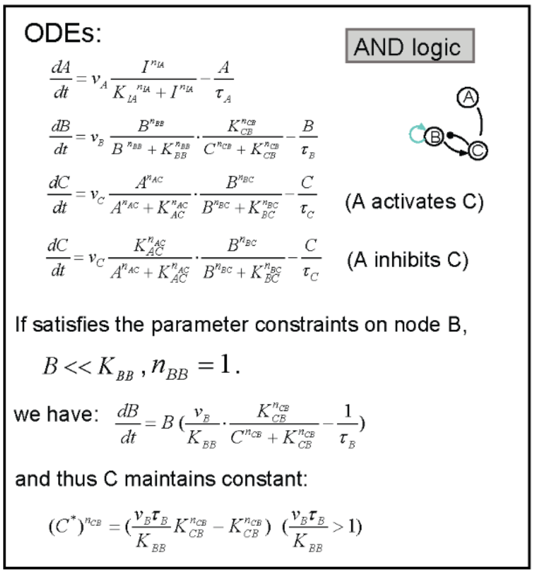

ODEs:

AND logic

$$\frac{dA}{dt} = v_A \frac{I^{n_{IA}}}{I^{n_{IA}} + K_{IA}^{n_{IA}}} - \frac{A}{\tau_A}$$

$$\frac{dB}{dt} = v_B \frac{B^{n_{BB}}}{B^{n_{BB}} + K_{BB}^{n_{BB}}} \cdot \frac{C^{n_{CB}}}{C^{n_{CB}} + K_{CB}^{n_{CB}}} - \frac{B}{\tau_B}$$

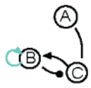

$$\frac{dC}{dt} = v_C \frac{A^{n_{AC}}}{A^{n_{AC}} + K_{AC}^{n_{AC}}} \cdot \frac{K_{BC}^{n_{BC}}}{B^{n_{BC}} + K_{BC}^{n_{BC}}} - \frac{C}{\tau_C}$$ (A activates C)

$$\frac{dC}{dt} = v_C \frac{K_{AC}^{n_{AC}}}{A^{n_{AC}} + K_{AC}^{n_{AC}}} \cdot \frac{K_{BC}^{n_{BC}}}{B^{n_{BC}} + K_{BC}^{n_{BC}}} - \frac{C}{\tau_C}$$ (A inhibits C)

If satisfies the parameter constraints on node B,

$$B << K_{BB}, n_{BB} = 1.$$

we have: $$\frac{dB}{dt} = B\left(\frac{v_B}{K_{BB}} \cdot \frac{C^{n_{CB}}}{C^{n_{CB}} + K_{CB}^{n_{CB}}} - \frac{1}{\tau_B}\right)$$

and thus C maintains constant:

$$(C^*)^{n_{CB}} = K_{CB}^{n_{CB}} \Big/ \left(\frac{\tau_B v_B}{K_{BB}} - 1\right) \quad \left(\frac{\tau_B v_B}{K_{BB}} > 1\right)$$

ODEs:

AND logic

$$\frac{dA}{dt} = v_A \frac{I^{n_{IA}}}{I^{n_{IA}} + K_{IA}^{n_{IA}}} \cdot \frac{B^{n_{BA}}}{B^{n_{BA}} + K_{BA}^{n_{BA}}} - \frac{A}{\tau_A}$$

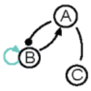

$$\frac{dB}{dt} = v_B \frac{B^{n_{BB}}}{B^{n_{BB}} + K_{BB}^{n_{BB}}} \cdot \frac{K_{AB}^{n_{AB}}}{K_{AB}^{n_{AB}} + A^{n_{AB}}} - \frac{B}{\tau_B}$$

$$\frac{dC}{dt} = v_C \frac{A^{n_{AC}}}{A^{n_{AC}} + K_{AC}^{n_{AC}}} - \frac{C}{\tau_C}$$ (A activates C)

$$\frac{dC}{dt} = v_C \frac{K_{AC}^{n_{AC}}}{A^{n_{AC}} + K_{AC}^{n_{AC}}} - \frac{C}{\tau_C}$$ (A inhibits C)

If satisfies the parameter constraints on node B,

$$B << K_{BB}, n_{BB} = 1.$$

we have: $$\frac{dB}{dt} = B\left(\frac{v_B}{K_{BB}} \cdot \frac{K_{AB}^{n_{AB}}}{K_{AB}^{n_{AB}} + A^{n_{AB}}} - \frac{1}{\tau_B}\right)$$

and thus A maintains constant:

$$(A^*)^{n_{AB}} = \left(\frac{v_B \tau_B}{K_{BB}} K_{AB}^{n_{AB}} - K_{AB}^{n_{AB}}\right) \quad \left(\frac{v_B \tau_B}{K_{BB}} > 1\right)$$

C maintains constant as it is a reporter of A.

ODEs:

AND logic

$$\frac{dA}{dt}=v_A\frac{I^{n_{IA}}}{K_{IA}^{n_{IA}}+I^{n_{IA}}}\cdot\frac{K_{BA}^{n_{BA}}}{B^{n_{BA}}+K_{BA}^{n_{BA}}}-\frac{A}{\tau_A}$$

$$\frac{dB}{dt}=v_B\frac{B^{n_{BB}}}{B^{n_{BB}}+K_{BB}^{n_{BB}}}\cdot\frac{A^{n_{AB}}}{K_{AB}^{n_{AB}}+A^{n_{AB}}}-\frac{B}{\tau_B}$$

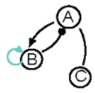

$$\frac{dC}{dt}=v_C\frac{A^{n_{AC}}}{A^{n_{AC}}+K_{AC}^{n_{AC}}}-\frac{C}{\tau_C}$$ (A activates C)

$$\frac{dC}{dt}=v_C\frac{K_{AC}^{n_{AC}}}{A^{n_{AC}}+K_{AC}^{n_{AC}}}-\frac{C}{\tau_C}$$ (A inhibits C)

If satisfies the parameter constraints on node B,

$B << K_{BB}, n_{BB}=1$.

we have: $$\frac{dB}{dt}=B\left(\frac{v_B}{K_{BB}}\cdot\frac{A^{n_{AB}}}{K_{AB}^{n_{AB}}+A^{n_{AB}}}-\frac{1}{\tau_B}\right)$$

and thus A maintains constant:

$$A^{*n_{AB}}=K_{AB}^{n_{AB}}\Big/\left(\frac{v_B\tau_B}{K_{BB}}-1\right)\quad\left(\frac{v_B\tau_B}{K_{BB}}>1\right)$$

C maintains constant as it is a reporter of A.

ODEs:

AND logic

$$\frac{dA}{dt}=v_A\frac{I^{n_{IA}}}{I^{n_{IA}}+K_{IA}^{n_{IA}}}\cdot\frac{B^{n_{BA}}}{B^{n_{BA}}+K_{BA}^{n_{BA}}}-\frac{A}{\tau_A}$$

$$\frac{dB}{dt}=v_B\frac{B^{n_{BB}}}{B^{n_{BB}}+K_{BB}^{n_{BB}}}\cdot\frac{C^{n_{CB}}}{C^{n_{CB}}+K_{CB}^{n_{CB}}}-\frac{B}{\tau_B}$$

$$\frac{dC}{dt}=v_C\frac{K_{AC}^{n_{AC}}}{A^{n_{AC}}+K_{AC}^{n_{AC}}}-\frac{C}{\tau_C}$$

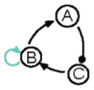

If satisfies the parameter constraints on node B,

$B << K_{BB}, n_{BB}=1$.

we have: $$\frac{dB}{dt}=B\left(\frac{v_B}{K_{BB}}\cdot\frac{C^{n_{CB}}}{C^{n_{CB}}+K_{CB}^{n_{CB}}}-\frac{1}{\tau_B}\right)$$

and thus C maintains constant:

$$(C^*)^{n_{CB}}=K_{CB}^{n_{CB}}\Big/\left(\frac{\tau_B v_B}{K_{BB}}-1\right)\quad\left(\frac{\tau_B v_B}{K_{BB}}>1\right)$$

ODEs:

AND logic

$$\frac{dA}{dt}=v_A\frac{I^{n_{IA}}}{K_{IA}^{n_{IA}}+I^{n_{IA}}}\cdot\frac{K_{BA}^{n_{BA}}}{B^{n_{BA}}+K_{BA}^{n_{BA}}}-\frac{A}{\tau_A}$$

$$\frac{dB}{dt}=v_B\frac{B^{n_{BB}}}{B^{n_{BB}}+K_{BB}^{n_{BB}}}\cdot\frac{C^{n_{CB}}}{C^{n_{CB}}+K_{CB}^{n_{CB}}}-\frac{B}{\tau_B}$$

$$\frac{dC}{dt}=v_C\frac{A^{n_{AC}}}{A^{n_{AC}}+K_{AC}^{n_{AC}}}-\frac{C}{\tau_C}$$

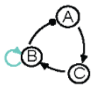

If satisfies the parameter constraints on node B,

$B << K_{BB}, n_{BB}=1$.

we have: $$\frac{dB}{dt}=B\left(\frac{v_B}{K_{BB}}\cdot\frac{C^{n_{CB}}}{C^{n_{CB}}+K_{CB}^{n_{CB}}}-\frac{1}{\tau_B}\right)$$

and thus C maintains constant:

$$(C^*)^{n_{CB}}=K_{CB}^{n_{CB}}\Big/\left(\frac{\tau_B v_B}{K_{BB}}-1\right)\quad\left(\frac{\tau_B v_B}{K_{BB}}>1\right)$$

ODEs:

AND logic

$$\frac{dA}{dt}=v_A\frac{I^{n_{IA}}}{I^{n_{IA}}+K_{IA}^{n_{IA}}}\cdot\frac{B^{n_{BA}}}{B^{n_{BA}}+K_{BA}^{n_{BA}}}-\frac{A}{\tau_A}$$

$$\frac{dB}{dt}=v_B\frac{B^{n_{BB}}}{B^{n_{BB}}+K_{BB}^{n_{BB}}}\cdot\frac{K_{CB}^{n_{CB}}}{K_{CB}^{n_{CB}}+C^{n_{CB}}}-\frac{B}{\tau_B}$$

$$\frac{dC}{dt}=v_C\frac{A^{n_{AC}}}{A^{n_{AC}}+K_{AC}^{n_{AC}}}-\frac{C}{\tau_C}$$

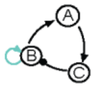

If satisfies the parameter constraints on node B,

$$B<<K_{BB},\ n_{BB}=1.$$

we have: $\frac{dB}{dt}=B(\frac{v_B}{K_{BB}}\cdot\frac{K_{CB}^{n_{CB}}}{K_{CB}^{n_{CB}}+C^{n_{CB}}}-\frac{1}{\tau_B})$

and thus C maintains constant:

$$(C^*)^{n_{CB}}=(\frac{v_B\tau_B}{K_{BB}}K_{CB}^{n_{CB}}-K_{CB}^{n_{CB}})\ \ (\frac{v_B\tau_B}{K_{BB}}>1)$$

ODEs:

AND logic

AND&OR logic

$$\frac{dA}{dt}=v_A\frac{I^{n_{IA}}}{K_{IA}^{n_{IA}}+I^{n_{IA}}}\cdot\frac{K_{BA}^{n_{BA}}}{B^{n_{BA}}+K_{BA}^{n_{BA}}}-\frac{A}{\tau_A}$$

$$\frac{dB}{dt}=v_B\frac{B^{n_{BB}}}{B^{n_{BB}}+K_{BB}^{n_{BB}}}\cdot\frac{K_{CB}^{n_{CB}}}{K_{CB}^{n_{CB}}+C^{n_{CB}}}-\frac{B}{\tau_B}$$

$$\frac{dC}{dt}=v_C\frac{K_{AC}^{n_{AC}}}{A^{n_{AC}}+K_{AC}^{n_{AC}}}-\frac{C}{\tau_C}$$

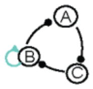

If satisfies the parameter constraints on node B,

$$B<<K_{BB},\ n_{BB}=1.$$

we have: $\frac{dB}{dt}=B(\frac{v_B}{K_{BB}}\cdot\frac{K_{CB}^{n_{CB}}}{K_{CB}^{n_{CB}}+C^{n_{CB}}}-\frac{1}{\tau_B})$

and thus C maintains constant:

$$(C^*)^{n_{CB}}=(\frac{v_B\tau_B}{K_{BB}}K_{CB}^{n_{CB}}-K_{CB}^{n_{CB}})\ \ (\frac{v_B\tau_B}{K_{BB}}>1)$$

ODEs:

AND logic

$$\frac{dA}{dt}=v_A\frac{I^{n_{IA}}}{K_{IA}^{n_{IA}}+I^{n_{IA}}}-\frac{A}{\tau_A}$$

$$\frac{dB}{dt}=v_B\frac{K_{AB}^{n_{AB}}}{A^{n_{AB}}+K_{AB}^{n_{AB}}}-\frac{B}{\tau_B}$$

$$\frac{dC}{dt}=v_C\frac{A^{n_{AC}}}{A^{n_{AC}}+K_{AC}^{n_{AC}}}\cdot\frac{B^{n_{BC}}}{B^{n_{BC}}+K_{BC}^{n_{BC}}}-\frac{C}{\tau_C}$$

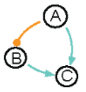

If satisfies the parameter constraints on node B,

$$A>>K_{AB}$$

we have: $v_B\tau_B\frac{K_{AB}^{n_{AB}}}{A^{*n_{AB}}}=B^*$

Substituting it into node C's equation. When

$A<<K_{AC}$ and $B<<K_{BC}$

at steady state, we have $C^*=v_C\tau_C\frac{A^{*n_{AC}}}{K_{AC}^{n_{AC}}}\cdot\frac{(v_B\tau_B\frac{K_{AB}^{n_{AB}}}{A^{*n_{AB}}})^{n_{BC}}}{K_{BC}^{n_{BC}}}$.

C maintains constant with $n_{AC}=n_{AB}n_{BC}$.

ODEs:

AND logic

AND&OR logic

$$\frac{dA}{dt} = v_A \frac{I^{n_{IA}}}{K_{IA}^{\ n_{IA}} + I^{n_{IA}}} - \frac{A}{\tau_A}$$

$$\frac{dB}{dt} = v_B \frac{A^{n_{AB}}}{A^{n_{AB}} + K_{AB}^{n_{AB}}} - \frac{B}{\tau_B}$$

$$\frac{dC}{dt} = v_C \frac{A^{n_{AC}}}{A^{n_{AC}} + K_{AC}^{n_{AC}}} \cdot \frac{K_{BC}^{n_{BC}}}{B^{n_{BC}} + K_{BC}^{n_{BC}}} - \frac{C}{\tau_C}$$

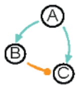

If satisfies the parameter constraints on node B,

$$A << K_{AB}$$

we have: $v_B \tau_B \frac{A^{*n_{AB}}}{K_{AB}^{n_{AB}}} = B^*$.

Substituting it into node C's equation. When

$A << K_{AC}$ and $B >> K_{BC}$

at steady state, we have $C^* = \frac{v_C \tau_C A^{*n_{AC}}}{(v_B \tau_B \frac{A^{*n_{AB}}}{K_{AB}^{n_{AB}}})^{n_{BC}}} \frac{K_{BC}^{n_{BC}}}{K_{AC}^{n_{AC}}}$.

C maintains constant with $n_{AC} = n_{AB} n_{BC}$.

ODEs:

AND logic

AND&OR logic

$$\frac{dA}{dt} = v_A \frac{I^{n_{IA}}}{K_{IA}^{\ n_{IA}} + I^{n_{IA}}} - \frac{A}{\tau_A}$$

$$\frac{dB}{dt} = v_B \frac{A^{n_{AB}}}{A^{n_{AB}} + K_{AB}^{n_{AB}}} - \frac{B}{\tau_B}$$

$$\frac{dC}{dt} = v_C \frac{K_{AC}^{n_{AC}}}{A^{n_{AC}} + K_{AC}^{n_{AC}}} \cdot \frac{B^{n_{BC}}}{B^{n_{BC}} + K_{BC}^{n_{BC}}} - \frac{C}{\tau_C}$$

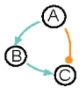

If satisfies the parameter constraints on node B,

$$A << K_{AB}$$

we have: $v_B \tau_B \frac{A^{*n_{AB}}}{K_{AB}^{n_{AB}}} = B^*$.

Substituting it into node C's equation. When

$A >> K_{AC}$ and $B << K_{BC}$

at steady state, we have $C^* = \frac{v_C \tau_C (v_B \tau_B \frac{A^{*n_{AB}}}{K_{AB}^{n_{AB}}})^{n_{BC}}}{A^{*n_{AC}}} \frac{K_{AC}^{n_{AC}}}{K_{BC}^{n_{BC}}}$.

C maintains constant with $n_{AC} = n_{AB} n_{BC}$.

ODEs:

AND logic

AND&OR logic

$$\frac{dA}{dt} = v_A \frac{I^{n_{IA}}}{K_{IA}^{\ n_{IA}} + I^{n_{IA}}} - \frac{A}{\tau_A}$$

$$\frac{dB}{dt} = v_B \frac{K_{AB}^{n_{AB}}}{A^{n_{AB}} + K_{AB}^{n_{AB}}} - \frac{B}{\tau_B}$$

$$\frac{dC}{dt} = v_C \frac{K_{AC}^{n_{AC}}}{K_{AC}^{n_{AC}} + A^{n_{AC}}} \cdot \frac{K_{BC}^{n_{BC}}}{K_{BC}^{n_{BC}} + B^{n_{BC}}} - \frac{C}{\tau_C}$$

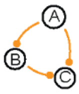

If satisfies the parameter constraints on node B,

$$A >> K_{AB}$$

we have: $v_B \tau_B \frac{K_{AB}^{n_{AB}}}{A^{*n_{AB}}} = B^*$

Substituting it into node C's equation. When

$A >> K_{AC}$ and $B >> K_{BC}$

at steady state, we have $C^* = v_C \tau_C \frac{K_{AC}^{n_{AC}}}{A^{*n_{AC}}} \cdot \frac{K_{BC}^{n_{BC}}}{(v_B \tau_B \frac{K_{AB}^{n_{AB}}}{A^{*n_{AB}}})^{n_{BC}}}$.

C maintains constant with $n_{AC} = n_{AB} n_{BC}$.

ODEs:

AND&OR logic

$$\frac{dA}{dt}=v_A\frac{I^{n_{IA}}}{K_{IA}^{n_{IA}}+I^{n_{IA}}}-\frac{A}{\tau_A}$$

$$\frac{dB}{dt}=v_B\frac{B^{n_{BB}}}{B^{n_{BB}}+K_{BB}^{n_{BB}}}\cdot\frac{K_{CB}^{n_{CB}}}{K_{CB}^{n_{CB}}+C^{n_{CB}}}-\frac{B}{\tau_B}$$

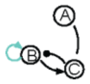

$$\frac{dC}{dt}=v_C\left(\frac{A^{n_{AC}}}{A^{n_{AC}}+K_{AC}^{n_{AC}}}+\frac{B^{n_{BC}}}{B^{n_{BC}}+K_{BC}^{n_{BC}}}\right)-\frac{C}{\tau_C}$$ (A activates C)

$$\frac{dC}{dt}=v_C\frac{K_{AC}^{n_{AC}}}{A^{n_{AC}}+K_{AC}^{n_{AC}}}\cdot\frac{B^{n_{BC}}}{B^{n_{BC}}+K_{BC}^{n_{BC}}}-\frac{C}{\tau_C}$$ (A inhibits C)

If satisfies the parameter constraints on node B,

$$B<<K_{BB},\, n_{BB}=1.$$

we have: $$\frac{dB}{dt}=B\left(\frac{v_B}{K_{BB}}\cdot\frac{K_{CB}^{n_{CB}}}{K_{CB}^{n_{CB}}+C^{n_{CB}}}-\frac{1}{\tau_B}\right)$$

and thus C maintains constant:

$$(C^*)^{n_{CB}}=\left(\frac{v_B\tau_B}{K_{BB}}K_{CB}^{n_{CB}}-K_{CB}^{n_{CB}}\right)\quad\left(\frac{v_B\tau_B}{K_{BB}}>1\right)$$

ODEs:

AND&OR logic

$$\frac{dA}{dt}=v_A\left(\frac{I^{n_{IA}}}{I^{n_{IA}}+K_{IA}^{n_{IA}}}+\frac{B^{n_{BA}}}{B^{n_{BA}}+K_{BA}^{n_{BA}}}\right)-\frac{A}{\tau_A}$$

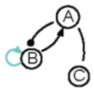

$$\frac{dB}{dt}=v_B\frac{B^{n_{BB}}}{B^{n_{BB}}+K_{BB}^{n_{BB}}}\cdot\frac{K_{AB}^{n_{AB}}}{K_{AB}^{n_{AB}}+A^{n_{AB}}}-\frac{B}{\tau_B}$$

$$\frac{dC}{dt}=v_C\frac{A^{n_{AC}}}{A^{n_{AC}}+K_{AC}^{n_{AC}}}-\frac{C}{\tau_C}$$ (A activates C)

$$\frac{dC}{dt}=v_C\frac{K_{AC}^{n_{AC}}}{A^{n_{AC}}+K_{AC}^{n_{AC}}}-\frac{C}{\tau_C}$$ (A inhibits C)

If satisfies the parameter constraints on node B,

$$B<<K_{BB},\, n_{BB}=1.$$

we have: $$\frac{dB}{dt}=B\left(\frac{v_B}{K_{BB}}\cdot\frac{K_{AB}^{n_{AB}}}{K_{AB}^{n_{AB}}+A^{n_{AB}}}-\frac{1}{\tau_B}\right)$$

and thus A maintains constant:

$$(A^*)^{n_{AB}}=\left(\frac{v_B\tau_B}{K_{BB}}K_{AB}^{n_{AB}}-K_{AB}^{n_{AB}}\right)\quad\left(\frac{v_B\tau_B}{K_{BB}}>1\right)$$

C maintains constant as it is a reporter of A.

ODEs:

AND&OR logic

$$\frac{dA}{dt}=v_A\left(\frac{I^{n_{IA}}}{I^{n_{IA}}+K_{IA}^{n_{IA}}}+\frac{B^{n_{BA}}}{B^{n_{BA}}+K_{BA}^{n_{BA}}}\right)-\frac{A}{\tau_A}$$

$$\frac{dB}{dt}=v_B\frac{B^{n_{BB}}}{B^{n_{BB}}+K_{BB}^{n_{BB}}}\cdot\frac{K_{CB}^{n_{CB}}}{K_{CB}^{n_{CB}}+C^{n_{CB}}}-\frac{B}{\tau_B}$$

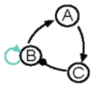

$$\frac{dC}{dt}=v_C\frac{A^{n_{AC}}}{A^{n_{AC}}+K_{AC}^{n_{AC}}}-\frac{C}{\tau_C}$$

If satisfies the parameter constraints on node B,

$$B<<K_{BB},\, n_{BB}=1.$$

we have: $$\frac{dB}{dt}=B\left(\frac{v_B}{K_{BB}}\cdot\frac{K_{CB}^{n_{CB}}}{K_{CB}^{n_{CB}}+C^{n_{CB}}}-\frac{1}{\tau_B}\right)$$

and thus C maintains constant:

$$(C^*)^{n_{CB}}=\left(\frac{v_B\tau_B}{K_{BB}}K_{CB}^{n_{CB}}-K_{CB}^{n_{CB}}\right)\quad\left(\frac{v_B\tau_B}{K_{BB}}>1\right)$$

ODEs:

Competitive Inhibition logic

$$\frac{dA}{dt}=v_A\frac{(\frac{I}{K_{IA}})^{n_{IA}}+(\frac{B}{K_{BA}})^{n_{BA}}}{(\frac{I}{K_{IA}})^{n_{IA}}+(\frac{B}{K_{BA}})^{n_{BA}}+1}-\frac{A}{\tau_A}$$

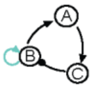

$$\frac{dB}{dt}=v_B\frac{(\frac{B}{K_{BB}})^{n_{BB}}}{(\frac{B}{K_{BB}})^{n_{BB}}+(\frac{C}{K_{CB}})^{n_{CB}}+1}-\frac{B}{\tau_B}$$

$$\frac{dC}{dt}=v_C\frac{(\frac{A}{K_{AC}})^{n_{AC}}}{(\frac{A}{K_{AC}})^{n_{AC}}+1}-\frac{C}{\tau_C}$$

If satisfies the parameter constraints on node B,

$B << K_{BB}\, , n_{BB} = 1\, .$

we have: $\frac{dB}{dt}=B(\frac{v_B}{K_{BB}}\cdot\frac{K_{CB}^{n_{CB}}}{C^{n_{CB}}+K_{CB}^{n_{CB}}}-\frac{1}{\tau_B})$

and thus C maintains constant:

$$(C^*)^{n_{CB}}=(\frac{v_B\tau_B}{K_{BB}}K_{CB}^{n_{CB}}-K_{CB}^{n_{CB}})\quad(\frac{v_B\tau_B}{K_{BB}}>1)$$

ODEs:

Competitive Inhibition logic

$$\frac{dA}{dt}=v_A\frac{(\frac{I}{K_{IA}})^{n_{IA}}}{(\frac{I}{K_{IA}})^{n_{IA}}+1}-\frac{A}{\tau_A}$$

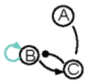

$$\frac{dB}{dt}=v_B(\frac{(\frac{B}{K_{BB}})^{n_{BB}}}{(\frac{B}{K_{BB}})^{n_{BB}}+(\frac{C}{K_{CB}})^{n_{CB}}+1})-\frac{B}{\tau_B}$$

$$\frac{dC}{dt}=v_C(\frac{(\frac{B}{K_{BC}})^{n_{BC}}+(\frac{A}{K_{AC}})^{n_{AC}}}{(\frac{B}{K_{BC}})^{n_{BC}}+(\frac{A}{K_{AC}})^{n_{AC}}+1})-\frac{C}{\tau_C}\quad\text{(A activates C)}$$

$$\frac{dC}{dt}=v_C(\frac{(\frac{B}{K_{BC}})^{n_{BC}}}{(\frac{B}{K_{BC}})^{n_{BC}}+(\frac{A}{K_{AC}})^{n_{AC}}+1})-\frac{C}{\tau_C}\quad\text{(A inhibits C)}$$

If satisfies the parameter constraints on node B,

$B << K_{BB}\, , n_{BB} = 1\, .$

we have: $\frac{dB}{dt}=B(\frac{v_B}{K_{BB}}\cdot\frac{K_{CB}^{n_{CB}}}{K_{CB}^{n_{CB}}+C^{n_{CB}}}-\frac{1}{\tau_B})$

and thus C maintains constant:

$$(C^*)^{n_{CB}}=(\frac{v_B\tau_B}{K_{BB}}K_{CB}^{n_{CB}}-K_{CB}^{n_{CB}})\quad(\frac{v_B\tau_B}{K_{BB}}>1)$$

ODEs: Competitive Inhibition logic

$$\frac{dA}{dt} = v_A \frac{(\frac{I}{K_{IA}})^{n_{IA}} + (\frac{B}{K_{BA}})^{n_{BA}}}{(\frac{I}{K_{IA}})^{n_{IA}} + (\frac{B}{K_{BA}})^{n_{BA}} + 1} - \frac{A}{\tau_A}$$

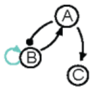

$$\frac{dB}{dt} = v_B (\frac{(\frac{B}{K_{BB}})^{n_{BB}}}{(\frac{B}{K_{BB}})^{n_{BB}} + (\frac{A}{K_{AB}})^{n_{AB}} + 1}) - \frac{B}{\tau_B}$$

$$\frac{dC}{dt} = v_C \frac{(\frac{A}{K_{AC}})^{n_{AC}}}{(\frac{A}{K_{AC}})^{n_{AC}} + 1} - \frac{C}{\tau_C}$$

If satisfies the parameter constraints on node B,

$$B << K_{BB} , n_{BB} = 1 .$$

we have: $\frac{dB}{dt} = B(\frac{v_B}{K_{BB}} \cdot \frac{K_{AB}^{n_{AB}}}{K_{AB}^{n_{AB}} + A^{n_{AB}}} - \frac{1}{\tau_B})$

and thus A maintains constant:

$$(A^*)^{n_{AB}} = (\frac{v_B \tau_B}{K_{BB}} K_{AB}^{n_{AB}} - K_{AB}^{n_{AB}}) \quad (\frac{v_B \tau_B}{K_{BB}} > 1)$$

C maintains constant as it is a reporter of A.

ODEs: Competitive Inhibition logic

$$\frac{dA}{dt} = v_A \frac{(\frac{I}{K_{IA}})^{n_{IA}}}{(\frac{I}{K_{IA}})^{n_{IA}} + 1} - \frac{A}{\tau_A}$$

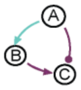

$$\frac{dB}{dt} = v_B \frac{(\frac{A}{K_{AB}})^{n_{AB}}}{(\frac{A}{K_{AB}})^{n_{AB}} + 1} - \frac{B}{\tau_B}$$

$$\frac{dC}{dt} = v_C (\frac{(\frac{B}{K_{BC}})^{n_{BC}}}{(\frac{B}{K_{BC}})^{n_{BC}} + (\frac{A}{K_{AC}})^{n_{AC}} + 1}) - \frac{C}{\tau_C}$$

If satisfies the parameter constraints on node B:

$$A << K_{AB}$$

we have: $v_B \tau_B \frac{A^{*n_{AB}}}{K_{AB}^{n_{AB}}} = B^*$.

Substituting it into node C's equation. When

$A >> K_{AC}$ or $B >> K_{BC}$

we have: $\frac{dC}{dt} = v_C (\frac{(\frac{B}{K_{BC}})^{n_{BC}}}{(\frac{B}{K_{BC}})^{n_{BC}} + (\frac{A}{K_{AC}})^{n_{AC}}}) - \frac{C}{\tau_C}$

at steady state, we have

$$C^* = v_C \tau_C \frac{(\frac{v_B \tau_B A^{*n_{AB}}}{K_{AB}^{n_{AB}} K_{BC}})^{n_{BC}}}{(\frac{v_B \tau_B A^{*n_{AB}}}{K_{AB}^{n_{AB}} K_{BC}})^{n_{BC}} + (\frac{A^*}{K_{AC}})^{n_{AC}}}$$

C maintains constant with $n_{AC} = n_{AB} n_{BC}$.

ODEs:

Competitive Inhibition logic

$$\frac{dA}{dt} = v_A \frac{(\frac{I}{K_{IA}})^{n_{IA}}}{(\frac{I}{K_{IA}})^{n_{IA}} + 1} - \frac{A}{\tau_A}$$

$$\frac{dB}{dt} = v_B \frac{(\frac{A}{K_{AB}})^{n_{AB}}}{(\frac{A}{K_{AB}})^{n_{AB}} + 1} - \frac{B}{\tau_B}$$

$$\frac{dC}{dt} = v_C (\frac{(\frac{A}{K_{AC}})^{n_{AC}}}{(\frac{B}{K_{BC}})^{n_{BC}} + (\frac{A}{K_{AC}})^{n_{AC}} + 1}) - \frac{C}{\tau_C}$$

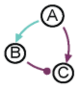

If satisfies the parameter constraints on node B:

$$A << K_{AB}$$

we have: $v_B \tau_B \frac{A^{*n_{AB}}}{K_{AB}^{n_{AB}}} = B^*$.

Substituting it into node C's equation. When

$A >> K_{AC}$ or $B >> K_{BC}$

we have: $\frac{dC}{dt} = v_C (\frac{(\frac{A}{K_{AC}})^{n_{AC}}}{(\frac{B}{K_{BC}})^{n_{BC}} + (\frac{A}{K_{AC}})^{n_{AC}}}) - \frac{C}{\tau_C}$

at steady state, we have

$$C^* = v_C \tau_C \frac{(\frac{A^*}{K_{AC}})^{n_{AC}}}{(\frac{v_B \tau_B A^{*n_{AB}}}{K_{AB}^{n_{AB}} K_{BC}})^{n_{BC}} + (\frac{A^*}{K_{AC}})^{n_{AC}}}$$

C maintains constant with $n_{AC} = n_{AB} n_{BC}$.

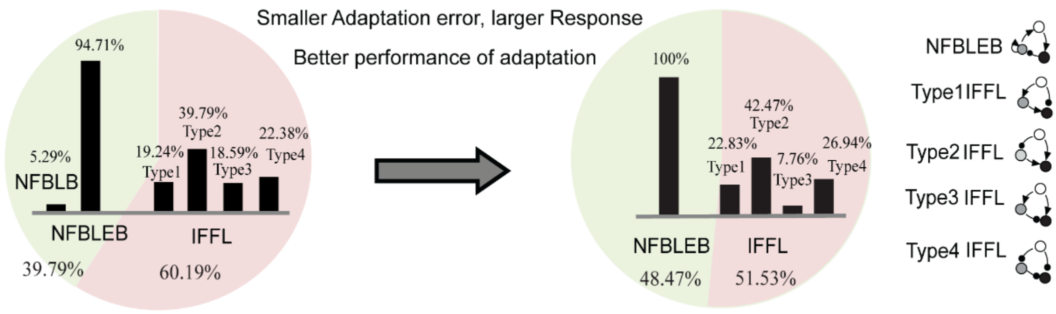

Figure S1. Motif categories maintain the same as the criteria for perfect adaptation changes. The compositions of every category converges on NFBLEB and four types of IFFL with more rigid criteria (AND logic). Left: adaptation error <0.01 with response >0.1, and right: adaptation error <0.005 with response >0.2.

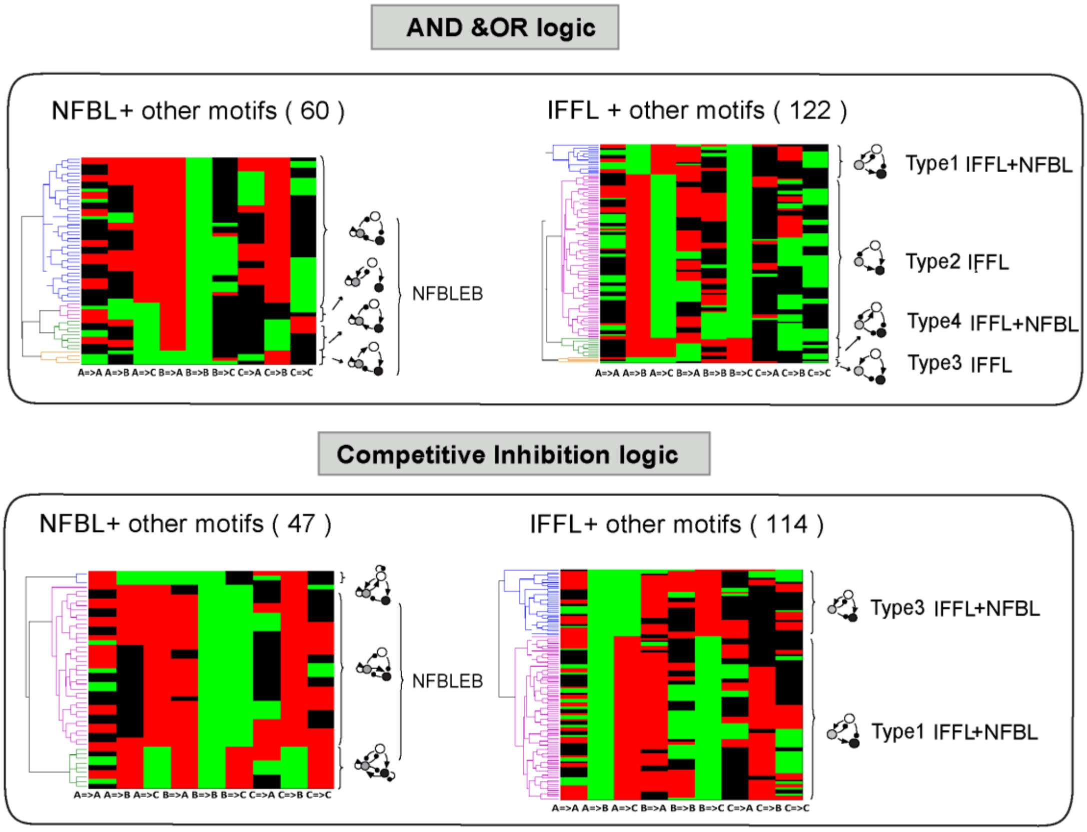

Figure S2. Clustering results of adaptive TRNs with AND&OR logic (up) and Competitive Inhibition logic (bottom). The network motifs associated with each of the sub-clusters are shown on the right.

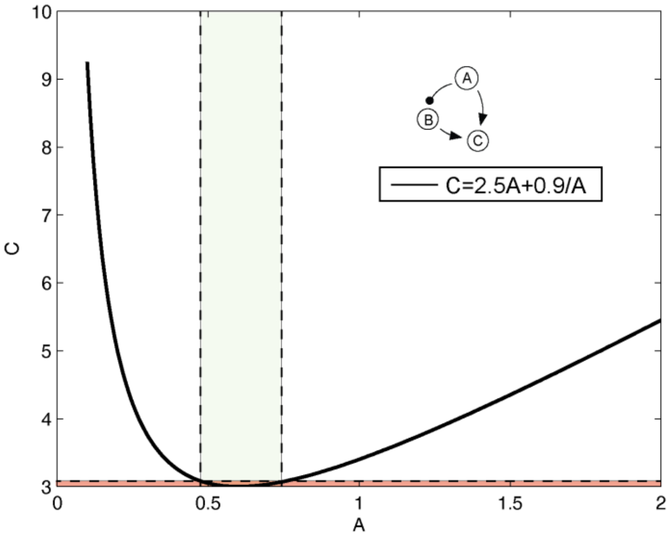

Figure S3. Type 2 IFFL adapts with a special condition with AND&OR logic.

References

1 Ma, W. Z., Lai, L. H., Qi, O. Y. & Tang, C. Robustness and modular design of the Drosophila segment polarity network. *Molecular Systems Biology* **2**, doi:Artn 70 10.1038/Msb4100111 (2006).

2 Iman, R.L., Davenport, J.M., and Zeigler, D.K.. Latin Hypercube Sampling (Program User's Guide) (Albuquerque, NM: Sandia Labs), pp. 77 (1980).
